# Methodological concerns regarding “Use of high-dose steroid therapy: addition of anakinra in the treatment of severe COVID-19”

**DOI:** 10.1590/1806-9282.20251818

**Published:** 2026-06-19

**Authors:** Fatih Yıldırım

**Affiliations:** 1 Çanakkale Mehmet Akif Ersoy State Hospital, Department of Rheumatology – Çanakkale, Turkey.

Dear Editor,

We read with great interest the article titled “Use of high-dose steroid therapy: addition of anakinra in the treatment of severe COVID-19,” recently published in your journal^1^. The study clearly reflects considerable clinical effort and dedication to understanding treatment approaches for severe COVID-19. However, several important methodological and statistical limitations may affect the validity and interpretation of its conclusions.

First, the hyperinflammatory state associated with COVID-19 differs from classical macrophage activation syndrome (MAS) in both clinical features and pathophysiology, as previously emphasized^2^. Labeling this hyperinflammatory state as MAS may lead to conceptual and clinical confusion, both in terms of nomenclature and diagnostic interpretation. The treatment of COVID-19–related hyperinflammatory states remains controversial, and no clear consensus has been reached in the literature regarding the efficacy of anti-cytokine therapies such as tocilizumab and anakinra. While early reports suggested that anakinra might improve hyperinflammation-related outcomes, subsequent systematic reviews could not confirm a mortality benefit^3,4^.

When considering the methodological aspects of the study, several issues warrant attention. At baseline, the anakinra+steroid group exhibited significantly higher ferritin levels and more severe computed tomography (CT) findings, introducing potential selection bias, which can substantially distort treatment effect estimates in retrospective analyses. Although this important limitation was acknowledged within the article, matching or adjustment for baseline severity (e.g., CT severity score or oxygenation indices) could have strengthened the methodological robustness and comparability between treatment arms^5^. Moreover, variables such as malignancy or underlying rheumatic disease, which may directly affect mortality, were not adjusted for in regression models. These patient subgroups should either have been excluded at baseline or incorporated into the regression models. Some patients had likely received varying doses of corticosteroids and additional antimicrobial agents before initiation of high-dose steroid (250 mg) and anakinra therapy; these pre-treatments may have influenced treatment-related complications and mortality, and a brief discussion of this issue would have provided greater clarity.

Another important issue concerns pre-treatment oxygenation parameters (e.g., SpO2, PaO2/FiO2, or need for high-flow oxygen). Although the limitations section of the article notes that the anak-inra+steroid group had poorer oxygenation status before treatment, these parameters were neither presented in the tables nor included in the multivariable analysis. These are crucial covariates in assessing 28-day mortality, and their inclusion in the multivariable analysis might have yielded different results. Likewise, while the duration of steroid therapy was found to be significant in univariate analysis, it was not included in the multivariate model, which could substantially confound the main outcome. Evaluating both the duration of steroid therapy and the total steroid exposure in the multivariable analysis could have provided more robust and reliable results. Lastly, mortality analysis based solely on proportions without survival methods (e.g., Kaplan-Meier or Cox models) limits the interpretability of time-dependent outcomes^6^.

In summary, the findings of this valuable study should be interpreted with caution in light of these limitations. Addressing the outlined methodological and statistical issues could significantly enhance the reliability and clinical applicability of the study’s conclusions.

I have no conflict of interest with the authors of the original article that is the subject of this letter.

## Data Availability

The datasets generated and/or analyzed during the current study are available from the corresponding author upon reasonable request.
